# Splenic Lesions in Dogs: A Retrospective Epidemiological and Pathological Study

**DOI:** 10.1155/vmi/9086933

**Published:** 2026-08-21

**Authors:** Hathali de las Mercedes Sanchez Mata, Janeth Agudelo Quintero, Diego Alfonso Aranzazu Taborda, David Alzate Velásquez, Julián David Muñoz Duque

**Affiliations:** ^1^ Quirón Pathobiology Research Group, School of Veterinary Medicine, Faculty of Agricultural Sciences, University of Antioquia (UdeA), Medellín, Colombia, udea.edu.co; ^2^ Professor, School of Veterinary Medicine and School of Agricultural and Livestock Production, University of Antioquia (UdeA), Medellín, Colombia, udea.edu.co; ^3^ Caninos y Felinos Veterinary Hospital, Medellín, Colombia

**Keywords:** hemangiosarcoma, splenic hematoma, splenic neoplasm, splenitis, splenomegaly

## Abstract

Splenic neoplastic and non‐neoplastic lesions are frequently diagnosed in dogs and often exhibit overlapping clinical signs and gross morphological features, rendering histopathological examination essential for definitive diagnosis, therapeutic planning, and prognostic assessment. In Colombia, epidemiological data regarding these lesions remain limited, whereas international studies report considerable heterogeneity in the relative frequencies of neoplastic and non‐neoplastic conditions. This study aimed to characterize the histopathological spectrum of splenic lesions in dogs and provide updated regional epidemiological data. A retrospective analysis was conducted on splenic specimens obtained from dogs subjected to total splenectomy between January 2019 and June 2024. All samples were processed and examined histopathologically at three veterinary pathology laboratories in Medellín, Colombia. Descriptive statistical analyses were performed considering year of submission, patient demographic data, morphological diagnosis, and concurrent histopathological findings. A total of 424 cases were evaluated, of which 282 (66.5%) corresponded to non‐neoplastic lesions and 142 (33.5%) to neoplastic processes. The mean age of dogs with non‐neoplastic lesions was 9.5 (< 1–16) years, whereas dogs with neoplastic lesions had a mean age of 10.2 (4–17) years. Splenitis was the most frequent non‐neoplastic diagnosis, accounting for 54.6% of cases and demonstrating a higher prevalence than reported in prior literature. Among malignant splenic neoplasms, hemangiosarcoma was the predominant diagnosis, representing 69.2% of cases. This study demonstrates a high prevalence of non‐neoplastic splenic lesions, particularly splenitis, and a frequent occurrence of multiple concurrent morphological diagnoses in non‐neoplastic cases. These findings provide novel regional epidemiological data and contribute original information to international literature, underscoring the importance of histopathological splenic evaluation for accurate diagnosis and clinical decision‐making.

## 1. Introduction

The spleen is the largest secondary lymphoid organ and plays a central role in systemic homeostasis through its involvement in immune defense, erythropoiesis, lymphopoiesis, erythrophagocytosis, and blood cell storage. Functionally, the white pulp contributes to antibody production and immune surveillance, whereas the red pulp filters senescent or damaged erythrocytes and serves as a reservoir for erythrocytes and platelets. Due to its physiological and anatomical characteristics, the spleen is susceptible to a wide spectrum of disorders, particularly in middle‐aged and geriatric dogs [1–4].

Splenic disorders are more frequently reported in large‐breed dogs, including Golden Retrievers, Labrador Retrievers, and German Shepherds, with body weight ≥ 21 kg identified as a significant risk factor [5–10]. Clinical signs are often nonspecific and include reduced appetite, lethargy, pallor, abdominal distension or pain, weight loss, vomiting, and diarrhea [2, 11, 12]. Dogs affected by splenic tumors, whether benign or malignant, may present with hemoperitoneum and anemia secondary to splenic rupture [13, 14]. In other instances, lesions are detected incidentally during imaging or surgical procedures [9].

The so‐called “two‐thirds rule,” originally described by Johnson et al. (1989) in dogs with splenomegaly, states that approximately two‐thirds of affected dogs have neoplastic disease and that two‐thirds of these neoplasms correspond to hemangiosarcoma (HSA) [7, 15–17]. Although this concept has subsequently been widely extrapolated to dogs with splenic masses in veterinary literature [14], its predictive value appears to vary according to clinical presentation and study population. Given the overlap in clinical presentation and gross appearance between non‐neoplastic and neoplastic lesions, histopathological examination remains essential for definitive diagnosis and appropriate clinical management [18, 19].

Histopathologically, splenic lesions in dogs are broadly classified as neoplastic and non‐neoplastic. Large studies conducted in North America and Europe have reported considerable variability in their relative prevalence, which is influenced by differences in study design and inclusion criteria. In Italy, a retrospective study including 682 canine spleens with and without hemoperitoneum found that non‐neoplastic lesions predominated (54.3%), with hematoma being the most frequent (57.0%) followed by nodular lymphoid hyperplasia (NLH) (37.3%). Neoplastic lesions accounted for 45.7% of cases, with HSA being the most prevalent neoplasm (54.5%), followed by other sarcomas (19.0%) and lymphoma (15.1%) [20].

In contrast, a prospective multicenter study from the United States including 345 dogs presenting with spontaneous hemoperitoneum secondary to a ruptured splenic mass reported a lower proportion of non‐neoplastic lesions (32.75%). In that cohort, nodular hyperplasia accounted for 89.38%, followed by myelolipoma (7.96%), whereas neoplasms represented 67.25% of cases, most commonly HSA (83.6%) and other sarcomas (10.34%) [13].

In Latin America, available data are limited and heterogeneous. In Brazil, non‐neoplastic lesions have been reported in 30%–64% of cases, with higher prevalence in smaller studies and lower prevalence in larger series [1, 10, 21–25]. In Argentina, 41% of cases were classified as such, whereas in Uruguay, only nine cases were described, providing insufficient data for characterization [26, 27]. A recent large study from Chile analyzing 507 samples found a predominance of non‐neoplastic lesions (67.5%), with hyperplastic conditions being the most frequent diagnoses [28].

In Colombia, epidemiological information on splenic lesions in dogs is scarce. A small study from Caldas reported non‐neoplastic alterations in 22.7% of dogs, benign neoplasms in 59%, and malignant neoplasms in 18.1% [29]. Comprehensive data linking these lesions to systemic diseases such as infectious, degenerative, or metabolic conditions are lacking. Moreover, although diagnostic capabilities have improved nationally, the clinical relevance of splenic lesions and standardized diagnostic protocols remain poorly defined. Descriptive studies, such as the present one, provide essential baseline data to inform future research and clinical practice.

Therefore, the present study aimed to characterize the frequency and histopathological spectrum of splenic lesions in dogs evaluated through three veterinary histopathology laboratories based in Medellín between 2019 and 2024. To our knowledge, this represents the first and one of the largest laboratory‐based studies on canine splenic pathology in Colombia.

## 2. Materials and Methods

### 2.1. Study Population

A retrospective review of histopathology reports describing splenic lesions in dogs was conducted. Cases were obtained from three veterinary histopathology laboratories based in Medellín, Antioquia, Colombia, between January 2019 and June 2024. These laboratories receive samples from multiple veterinary clinics and hospitals within the region. All samples consisted of spleen fragments obtained by total splenectomy. Incisional biopsies were excluded.

### 2.2. Data Collection

Pathology submission forms and corresponding histopathology reports were reviewed. Extracted data included the year of the report (2019–2024), animal demographics (breed, age, and sex), clinical signs, presence of hemoabdomen, and gross lesion pattern. Macroscopic presentations were classified into four categories: single mass, multiple masses, splenomegaly, and nodular pattern. A single mass was defined as one splenic lesion > 1 cm in diameter and multiple masses as more than one lesion > 1 cm. Splenomegaly was defined as diffuse enlargement of the spleen without formation of a nodule or mass, whereas the nodular pattern referred to lesions < 1 cm in diameter.

Lesions were categorized as non‐neoplastic or neoplastic. Splenitis cases within the non‐neoplastic group were further subclassified according to established histopathological criteria for inflammatory lesions. Neoplastic cases were subsequently classified according to biological behavior as malignant or benign.

Histopathological diagnoses were recorded following standard morphological criteria. In non‐neoplastic cases, more than one diagnosis could be present within the same case; therefore, all reported histopathological diagnoses were recorded independently without hierarchical prioritization. Up to three diagnoses per case were documented when applicable. For neoplastic lesions, each case was assigned a single diagnosis based on the pathologist’s report.

Common associated histopathological findings, including necrosis, hemorrhage, extramedullary hematopoiesis, inflammation, hematoma, siderotic plaques, lymphoid tissue changes (alterations in the splenic white pulp, including lymphoid hyperplasia, atrophy, and depletion), and central arteriolar thickening, were recorded when documented in the original histopathology reports.

All histopathological evaluations were performed by board‐certified veterinary pathologists at the participating diagnostic laboratories.

### 2.3. Histological Processing and Staining

Splenic samples were fixed and processed following standard histopathological protocols routinely used in the participating diagnostic laboratories. Tissues were fixed in 10% neutral buffered formalin, embedded in paraffin, sectioned at 4–5 μm, and stained with hematoxylin and eosin (H&E) for microscopic evaluation. No immunohistochemical procedures were performed; only H&E‐stained sections were reviewed for this study.

### 2.4. Data Analysis

Data were entered into Excel (Microsoft Corp., Redmond, WA, USA) and analyzed using SAS software Version 9.4 (SAS Institute Inc., Cary, NC, USA). Descriptive statistics were used to summarize the data. Categorical variables were expressed as absolute and relative frequencies, whereas continuous variables were summarized as mean (range). Normality was assessed using the Shapiro–Wilk test. Age was analyzed both as a continuous variable and grouped into categories (< 5, 5–8, 9–12, and > 12 years) to allow comparative analysis. Dogs younger than 1 year (*n* = 5) were included in categorical analyses but excluded from mean age calculations because exact age in months was unavailable.

Associations between categorical variables were evaluated using chi‐square or Fisher’s exact tests, as appropriate. Odds ratios (ORs) and corresponding 95% confidence intervals (95% CI) were calculated using cases with complete data for the variable of interest. All statistical tests were two‐sided, and statistical significance was set at *p* < 0.05.

### 2.5. Ethical Considerations

This study analyzed data obtained during routine histopathological diagnosis. Prior informed consent was obtained from pet owners for academic and scientific use of data, and laboratories authorized access to records. Ethical committee approval was deemed unnecessary, as no additional interventions were performed on animals.

## 3. Results

### 3.1. Study Population

A total of 424 canine splenectomy cases were included. All samples consisted of splenic tissue fragments submitted for histopathological evaluation between January 2019 and June 2024. The annual case distribution was 82 cases (2019), 47 (2020), 60 (2021), 86 (2022), 113 (2023), and 36 during the first half of 2024. Most submissions originated from the department of Antioquia (92%), with additional cases from two other Colombian departments (Atlántico and Norte de Santander).

The study population comprised 222 males (52.4%) and 202 females (47.6%). Age information was unavailable in six cases, and five dogs were younger than 1 year of age. The mean age (range) of dogs with non‐neoplastic lesions was 9.5 (< 1–16) years, whereas dogs with neoplastic lesions had a mean age of 10.2 years [4–17]. Dogs aged 9–12 years showed significantly increased odds of splenic neoplasia compared with other age groups (OR: 1.85; 95% CI: 1.21–2.81; *p* *= *0.004).

A total of 45 breeds were represented. Crossbreed dogs were the most frequent (26.7%), followed by Schnauzer (7.3%), Pit Bull Terrier (6.6%), Beagle (6.1%), Golden Retriever (5.4%), Bulldog (5.4%), Labrador Retriever (5%), and Poodle (4%).

### 3.2. Clinical Presentation

Clinical information was obtained from histopathology submission forms. This section was categorized based on reported clinical signs, whereas hemoperitoneum was evaluated as a separate clinical variable.

Abdominal distension and/or abdominal pain were the most frequently reported clinical signs (41%, 174/424). Splenic lesions were detected incidentally by ultrasonography in 33.7% of cases (143/424). Dogs with incidental findings were more likely to have non‐neoplastic lesions compared with those with non‐incidental presentations (OR: 1.75; 95% CI: 1.10–2.80; *p* *= *0.019).

Other nonspecific clinical signs, including lethargy, fever, weight loss, and reduced appetite, were reported in 10.6% (45/424). In 62 cases (14.6%), clinical information was not available in the submission forms, precluding further classification.

Hemoperitoneum was reported in 12.7% (54/424) of dogs and was significantly associated with splenic neoplasia (OR: 3.92; 95% CI: 2.15–7.13; *p* < 0.001), particularly HSA (OR: 8.83; 95% CI: 4.70–16.58; *p* < 0.001).

### 3.3. Lesion Types

**FIGURE 1:**
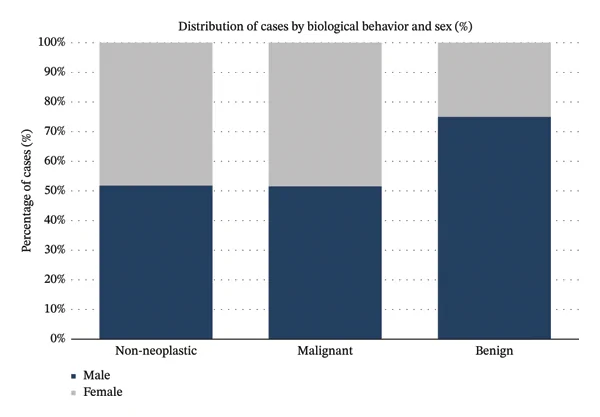
Distribution of cases by biological behavior and sex (%). *Note:* Bars represent the proportion of males and females within each lesion category. Percentages were calculated relative to the total number of dogs in each diagnostic group. Non‐neoplastic lesions include inflammatory, reactive, and degenerative conditions, whereas neoplastic lesions include benign and malignant splenic tumors.

Of the 424 cases, 282 (66.5%) were classified as non‐neoplastic lesions, whereas 142 (33.5%) corresponded to splenic neoplasms. Among neoplastic cases, malignant behavior predominated (130/142; 91.5%), whereas benign neoplasms accounted for 8.5% (12/142) (Figure 1).

### 3.4. Macroscopic Findings

Mass‐forming lesions were the predominant macroscopic presentation. A single splenic mass was the most frequent pattern (256/424; 60.4%), followed by multiple masses (76/424; 17.9%), splenomegaly (42/424; 9.9%), and nodular pattern (11/424; 2.6%). In 39 dogs (9.2%), the macroscopic pattern was not specified.

No significant association was found between macroscopic pattern and final diagnosis (non‐neoplastic vs. neoplastic) (*χ* ^2^ = 7.9, *p* *= *0.10). However, analysis of individual macroscopic patterns revealed several significant associations. Dogs presenting a single splenic mass were more frequently incidentally detected (OR: 1.67; 95% CI: 1.04–2.69; *p* *= *0.03) and this presentation was also associated with NLH (OR: 4.04; 95% CI: 1.86–8.77; *p* = 0.003), and splenic hematoma (OR: 2.48; 95% CI: 1.34–4.60; *p* = 0.003).

Conversely, dogs with multiple splenic masses showed increased odds of malignancy (OR: 1.76; 95% CI: 1.04–2.96; *p* = 0.03), and HSA (OR: 2.08; 95% CI: 1.19–3.65; *p* = 0.01). Additional associations are summarized in Table 1.

**TABLE 1:** Summary of significant clinicopathological associations identified in dogs undergoing splenectomy.

Variable	Outcome	OR	95% CI	*p* value
Demographic variables				
Sex (male vs. female)	Neoplasia	1.07	0.72–1.61	0.73
	HSA	1.05	0.66–1.68	0.93
	Splenitis	0.74	0.49–1.11	0.15
Age (9–12 years vs. other age groups)	Neoplasia	1.85	1.21–2.81	**0.004**
	HSA	1.99	1.21–3.26	**0.009**
	Splenitis	0.83	0.56–1.24	0.36
Crossbreed vs. purebred	HSA	1.61	0.97–2.65	0.083

Clinical presentation				
Incidental detection	Non‐neoplasm	1.75	1.10–2.80	**0.019**
	Single mass	1.67	1.04–2.69	**0.033**
	Multiple masses	1.33	0.78–2.26	0.296
Hemoperitoneum (present vs. absent)	Neoplasia	3.92	2.15–7.13	**< 0.001**
	HSA	8.83	4.70–16.58	**< 0.001**
	Lymphoma	0.31	0.04–2.30	0.24
	Splenitis	0.41	0.21–0.80	**0.009**
	NLH	0.19	0.04–0.82	**0.027**

Non‐hemoperitoneum HSA				
Incidental detection	HSA	0.81	0.38–1.75	0.590
Abdominal distension and/or pain	HSA	1.17	0.53–2.58	0.690

Macroscopic findings				
Single mass (present vs. absent)	Malignant	0.79	0.50–1.24	0.296
	Splenitis	0.77	0.50–1.18	0.23
	Splenic hematoma	2.48	1.34–4.60	**0.003**
	NLH	4.04	1.86–8.77	**< 0.001**
Multiple masses (present vs. absent)	Malignant	1.76	1.04–2.96	**0.032**
	Splenitis	0.77	0.46–1.29	0.32
	HSA	2.08	1.19–3.65	0.010

Histopathological findings				
Splenic hematoma^∗^	HSA	1.45	0.90–2.33	0.13

*Note:* Odds ratios were calculated using 2 × 2 contingency tables. Cases with missing data were excluded from the association analyses. Values in bold indicate statistical significance.

Abbreviations: CI, confidence interval; HSA, hemangiosarcoma; NLH, nodular lymphoid hyperplasia; OR, odds ratio.

^∗^The variable refers to the presence of splenic hematoma as a concurrent histopathological finding rather than as a primary non‐neoplastic diagnosis.

### 3.5. Non‐Neoplastic Splenic Lesions

Non‐neoplastic lesions represented 66.5% of cases (282/424). Because multiple morphological processes could occur within the same spleen, entities were not mutually exclusive; therefore, up to three diagnoses were recorded per case, yielding a cumulative total of 451 non‐neoplastic diagnoses.

**FIGURE 2:**
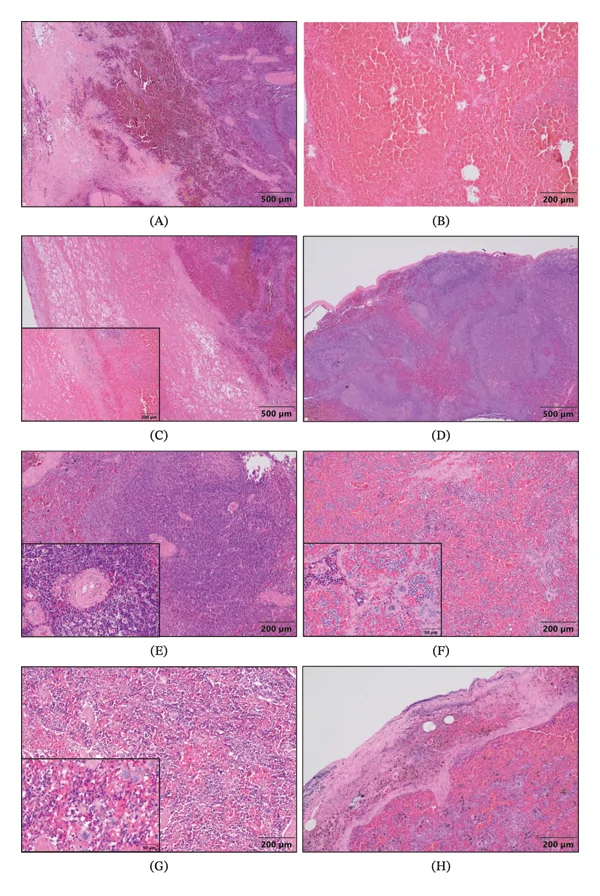
Histopathological non‐neoplastic splenic features using hematoxylin and eosin stain. (A) *Chronic hemorrhagic splenitis* (4×): hemorrhage accompanied by neutrophilic infiltration, hemosiderin deposits, and areas of fibrosis. (B) *Non-organized splenic hematoma* (10×): presence of abundant erythrocytes and minimal cellular debris, supported by fibrin. (C) *Organized splenic hematoma* (4×/10×): hemorrhagic lesion characterized by fibrin deposition supporting erythrocytes, cellular debris, hemosiderin accumulation, and collagen deposition. (D) *Nodular lymphoid hyperplasia* (4×): circumscribed proliferation of lymphocytes organized into lymphoid follicles, occasionally with coalescence. (E) *Hypertrophy of the central arteriole* (10×/40×): in a dog with lymphoid nodular hyperplasia. (F–G) *Severe extramedullary hematopoiesis* (10×/40×): abundant erythroid islands and megakaryocytes were found. (H) *Subcapsular hemorrhage and siderotic nodules* (10×): extensive subcapsular hemorrhage and siderosclerosis, accompanied by mild fibroplasia and EMH.

Inflammatory and reactive conditions predominated. Splenitis was the most frequent diagnosis (54.6%, 154/282 dogs) (Figure 2A), followed by splenic hematoma (33.7%, 95/282) (Figure 2B,C) and NLH (22.3%, 63/282) (Figure 2D,E). The main non‐neoplastic diagnoses and their clinicopathological characteristics are summarized in Table 2. Detailed demographic features, concurrent diagnoses, and associated histopathological findings for each morphological diagnosis are presented in Supporting Table S1.

**TABLE 2:** Main non‐neoplastic splenic diagnoses in dogs undergoing splenectomy and their clinicopathological characteristics (*n* = 282).

Non‐neoplastic diagnoses	Cases *n* (%)	Median age years (range)	Sex (F/M)	Most affected breeds	Common macroscopic pattern	Common concurrent morphological diagnosis	Common concurrent histopathological findings
Splenitis	154 (54.6)	9.5 (< 1–16)^a^	81/73	Crossbreed, Beagle, Pit Bull Terrier	Single mass	Splenic hematoma, nodular lymphoid hyperplasia (NLH)	Extramedullary hematopoiesis (EMH), hemorrhage
Splenic hematoma	95 (33.7)	10.1 (4–16)^b^	37/41	Crossbreed, Pit Bull Terrier	Single mass	Splenitis	Hemorrhage, EMH
Nodular lymphoid hyperplasia (NLH)	63 (22.3)	8.9 (< 1–15)^c^	28/35	Crossbreed, Poodle, Shih Tzu	Single mass	Splenitis, splenic hematoma	Hemorrhage, EMH, inflammation
Extramedullary hematopoiesis (EMH)	30 (10.6)	9.6 (< 1–15)^d^	17/13	Crossbreed, Poodle, Pit Bull Terrier, Bulldog	Single mass	Splenitis, splenic hematoma, NLH	Lymphoid tissue changes^∗^, hemorrhage, inflammation
Siderotic nodules	27 (9.6)	8.4 (1–14)	12/15	Crossbreed, Bulldog	Single mass	Splenitis, splenic hematoma, EMH, NLH, splenic hemorrhage	EMH, hemorrhage, inflammation, lymphoid tissue changes

*Note:* Because multiple morphological processes may occur within the same spleen, diagnoses were not mutually exclusive, and up to three histological diagnoses could be recorded per case. The complete distribution of non‐neoplastic lesions is provided in Supporting Table S1.

Abbreviations: EMH, extramedullary hematopoiesis; NLH, nodular lymphoid hyperplasia.

^∗^Lymphoid tissue changes include white pulp alterations such as hyperplasia, atrophy, or depletion when reported.

^a^In three cases, age was not reported, and one dog was < 1 year old.

^b^Age was not reported in one case.

^c^In two cases, age was not reported, and one dog was < 1 year old.

^d^Age was not reported in one case, and one dog was < 1 year old.

Among inflammatory patterns, hemorrhagic splenitis was the most frequent subtype (46.8%, 72/154), followed by congestive (22.7%, 35/154) and chronic/active splenitis (18.2%, 28/154). The remaining patterns were less common, each representing less than 5% of cases (Table 3). Dogs presenting hemoperitoneum had significantly lower odds of splenitis (OR: 0.41; 95% CI: 0.21–0.81; *p* = 0.009).

**TABLE 3:** Distribution of histopathological inflammatory patterns observed in canine splenitis (*n* = 154).

Inflammatory pattern	Cases *n*	Splenitis %
Hemorrhagic	72	46.8
Congestive	35	22.7
Chronic/active	28	18.2
Fibrosing	6	3.9
Suppurative	5	3.2
Mixed cases^∗^	4	2.6
Granulomatous/pyogranulomatous	2	1.3
Necrotic	2	1.3
**Total**	**154**	**100.0**

*Note:* Bold values indicate the total number of splenitis cases and the corresponding percentage.

^∗^Mixed cases corresponded to hemorrhagic splenitis accompanied by additional inflammatory descriptors (e.g., congestive, fibrosing, or chronic changes).

NLH most commonly presented as a solitary mass, consistent with the significant association between this diagnosis and single‐mass presentation described above. However, NLH was not significantly associated with hemoperitoneum (OR: 0.19; 95% CI: 0.04–0.82; *p* = 0.027).

Co‐occurrence among the most common diagnoses was frequent. Splenitis overlapped with splenic hematoma in 28.6% of affected dogs (44/154), whereas concurrent NLH was identified in 10.4% (16/154).

Consistent secondary histopathological lesions were observed across the major diagnoses. Hemorrhage and extramedullary hematopoiesis were the most frequent accompanying findings, particularly in cases of splenitis (79.9% and 83.1%, respectively), splenic hematoma (84.2% and 68.4%), and NLH (65.0% and 54.0%). Detailed secondary histopathological findings are provided in Supporting Table S2.

Other primary diagnoses collectively represented a minor proportion of cases (Supporting Table S1).

### 3.6. Neoplastic Splenic Lesions

Splenic neoplasms accounted for 33.5% of cases (142/424 dogs), of which 91.5% (130/142) exhibited malignant behavior (Table 4). Tumor grading was not systematically applied due to the limited availability and inconsistent validation of grading systems for canine splenic neoplasms [30]. Detailed demographic characteristics, macroscopic presentation, concurrent diagnoses, and associated histopathological findings for each neoplasm are presented in Supporting Table S3.

**TABLE 4:** Main neoplastic splenic diagnoses in dogs undergoing splenectomy and their clinicopathological characteristics (*n* = 142).

Neoplastic diagnoses	Cases n (%)	Median age years (range)	Sex (F/M)	Most affected breeds	Common macroscopic pattern	Common concurrent histopathological findings
Hemangiosarcoma (HSA)^a^	90 (63.4)^b^	10.6 (5–17)	42/48	Crossbreed, Schnauzer	Single mass	Hemorrhage, necrosis, splenic hematoma, inflammation
Lymphoma^c^	20 (14.1)	9.3 (4–14)	11/9	Crossbreed, Bulldog, Rottweiler	Splenomegaly	Lymphoid tissue changes^∗^, hemorrhage, necrosis
Undifferentiated sarcoma^d^	8 (5.6)	10.0 (7–12)	3/5	Pit Bull	Single mass	Hemorrhage, inflammation, necrosis
Myelolipoma	8 (5.6)	10.0 (8–13)	2/6	Labrador Retriever, Crossbreed	Single mass	Inflammation, extramedullary hematopoiesis (EMH), hemorrhage, lymphoid tissue changes

*Note:* Detailed distribution of all neoplastic diagnoses and associated findings is provided in Supporting Table S2.

Abbreviations: EMH, extramedullary hematopoiesis; HSA, hemangiosarcoma.

^∗^Lymphoid tissue changes include white pulp alterations such as hyperplasia, atrophy, depletion, or neoplasia when reported.

^a^Three cases were consistent with hemangiosarcoma and require more specific tests to confirm the diagnosis.

^b^In two cases, the age was not reported.

^c^One case was consistent with lymphoma and required more specific tests to confirm the diagnosis.

^d^Cases previously diagnosed as fibrohistiocytic nodules (*n* = 3) and malignant fibrous histiocytoma (*n* = 1) were reclassified as undifferentiated sarcoma according to current classification criteria. Immunohistochemistry was not available in this study.

Concurrent diagnoses were less frequent in neoplastic cases and primarily corresponded to reactive or degenerative non‐neoplastic changes. In contrast to non‐neoplastic lesions, neoplastic diagnoses were mutually exclusive, and no dog presented more than one primary splenic neoplasm.

**FIGURE 3:**
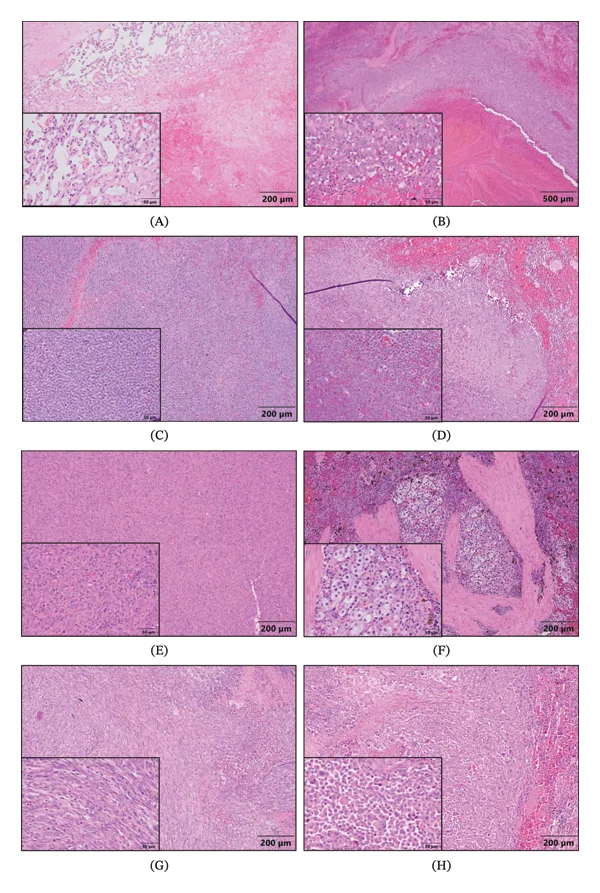
Histopathological neoplastic splenic features using hematoxylin and eosin stain. (A) *Well-differentiated hemangiosarcoma* (10×/40×): composed of spindle‐shaped cells arranged in vascular spaces containing erythrocytes; cells exhibit oval, hyperchromatic nuclei, moderate anisokaryosis, and fibrillar cytoplasm. (B) *Poorly differentiated hemangiosarcoma* (4×/40×): composed of cells with moderate pleomorphism arranged in a solid pattern or irregular channels containing erythrocytes. This neoplasm is surrounded by an extensive hematoma: spindle‐shaped and ballooniform cells with oval nuclei and cytoplasm exhibiting indistinct cell borders. (C) *Small cell lymphoma* (10×/40×): a neoplasm consisting of round cells, diffusely distributed and supported by scant fibrous stroma: small cells with marked atypia, spherical, euchromatic nuclei, prominent nucleoli, moderate anisokaryosis, and scant cytoplasm. (D) *Follicular lymphoma* (10×/40×): neoplastic lymphocytes organized into follicles: medium‐sized cells with spherical, euchromatic nuclei containing 1 to 3 prominent nucleoli, moderate anisokaryosis, and scant cytoplasm. (E) *Non-angiogenic, non-lymphogenic sarcoma* (10×/40×): a mesenchymal neoplasm exhibiting a solid pattern, with histological origin yet to be clarified: cells displaying marked pleomorphism and anisokaryosis, variable chromatin, prominent nucleoli, and variable eosinophilic cytoplasm. (F) *Undifferentiated neoplasm* (10×/40×): focus of non‐encapsulated neoplasia with a pseudoacinar arrangement, bordered by areas of hemorrhage and fibroplasia: cells with large nuclei containing a prominent central nucleolus, vacuolated to fibrillar cytoplasm with relatively well‐defined cell borders. Histological features did not allow definitive determination of histogenetic origin based on routine histology alone. (G) *Fibrosarcoma* (10×/40×): abundant fusiform cells organized in interwoven fascicles: spindle‐shaped cells with oval to elongated, euchromatic nuclei, moderate anisokaryosis, and fibrillar cytoplasm. (H) *Histiocytic sarcoma* (10×/40×): organized in a diffuse pattern of large cells with abundant cytoplasm, marked atypia, and a high degree of pleomorphism in mononuclear cells: frequent presence of multinucleated giant cells.

Among malignant neoplasms, HSA (Figure 3A,B) predominated (69.2%, 90/130). Dogs aged 9–12 years showed significantly increased odds of developing this neoplasm (OR: 1.99; 95% CI: 1.21–3.26; *p* = 0.009). Hemoperitoneum and multiple splenic masses were also significantly associated with HSA, as described above. However, among dogs with available clinical information, 57.9% (44/76) of HSA cases did not present with abdominal bleeding at diagnosis. Within this subgroup, splenic lesions were most commonly detected incidentally by ultrasonography (38.6%, 17/44), whereas others presented with abdominal distension and/or pain (34.1%, 15/44). Additional nonsignificant clinical and demographic associations are summarized in Table 1. Lymphoma ranked second (Figure 3C,D), accounting for 15.4% (20/130) of malignant tumors.

Consistent secondary histopathological lesions were observed across the major malignant neoplasms. Hemorrhage and necrosis were the most frequent, particularly in cases of HSA (78.9% and 56.7%, respectively) and lymphoma (65.0% and 40.0%). Histological features consistent with splenic hematoma were identified in 40.0% (36/90) of HSA cases compared with 31.4% (105/334) of non‐HSA cases; however, this difference was not statistically significant (OR: 1.45; 95% CI: 0.90–2.33; *p* = 0.13). Detailed secondary histopathological findings are provided in Supporting Table S4.

The remaining malignant neoplasms collectively accounted for 15.4% (20/130) and included undifferentiated sarcomas (Figure 3E–F), followed by fibrosarcoma (Figure 3G), histiocytic sarcoma (Figure 3H), liposarcoma, myxosarcoma, and a single mast cell tumor. Three cases originally described as fibrohistiocytic nodules and one case reported as malignant fibrous histiocytoma were reclassified as undifferentiated sarcomas according to contemporary veterinary tumor classification criteria [31] and the absence of immunohistochemical characterization. Additionally, one case originally diagnosed as an undifferentiated neoplasm was included within this category for descriptive purposes, although its definitive lineage could not be established. Benign neoplasms represented 8.5% of splenic tumors (12/142). Myelolipoma was the most frequent benign tumor (66.7%, 8/12), followed by plasmacytoma (25.0%, 3/12) and cavernous hemangioma (8.3%, 1/12).

## 4. Discussion

Splenic disorders in dogs encompass a wide spectrum of lesions with diverse etiologies and biological behaviors [2, 15, 18]. The increasing detection of these conditions is likely related to the widespread use of advanced diagnostic imaging techniques, particularly ultrasonography [32, 33], together with cytological [3, 34, 35] and histopathological evaluation. In the present study, a large retrospective histopathological evaluation of splenectomized dogs was performed to better characterize the epidemiological and pathological patterns of splenic disease in a regional population.

Three principal findings emerged from this study. First, non‐neoplastic lesions were more frequent than neoplastic processes in dogs undergoing splenectomy. Second, splenitis represented an unexpectedly high proportion of non‐neoplastic diagnoses. Third, multiple concurrent histopathological diagnoses were frequently identified within the same spleen, highlighting the complex pathological processes that may occur simultaneously in this organ.

The relative proportion of neoplastic and non‐neoplastic splenic lesions has been widely discussed in veterinary literature and is often summarized by the so‐called “two‐thirds rule,” originally described in dogs with splenic enlargement in a mixed surgical and postmortem population [7]. Although this concept has subsequently been broadly applied to dogs with splenic masses, later studies have shown that its predictive value varies considerably according to case selection, clinical presentation, and inclusion criteria.

In dogs presenting with hemoperitoneum secondary to splenic mass rupture, neoplasia has been reported in 67.2% (232/345) of cases, of which 83.6% (194/232) were HSA [13]. Similarly, another large retrospective study reported HSA in 87.3% (733/840) of malignant splenic lesions, although this proportion may be overestimated because benign neoplasms were not included in that analysis [36]. In contrast, studies evaluating incidentally detected nonruptured splenic nodules/masses or splenomegaly have reported a substantially lower proportion of neoplastic disease, with neoplasia accounting for 32.4% (34/105) and 44.8% (87/194) of cases, respectively. In those studies, HSA represented 52.9% (18/34) and 50.6% (44/87) of neoplasms [9, 37].

In the present study, neoplastic lesions represented 33.5% (142/424) of cases, which is considerably lower than proportions reported in studies focusing exclusively on ruptured splenic masses. This difference is likely explained by the heterogeneous nature of our study population, which included dogs with a variety of clinical and macroscopic presentations. Therefore, direct comparisons with studies restricted to dogs with hemoperitoneum should be interpreted cautiously.

Among neoplastic processes, HSA was the most frequent diagnosis, representing 63.4% (90/142), consistent with previous reports identifying HSA as the most common splenic neoplasia in dogs [5, 38–42]. The reported prevalence of HSA varies widely across studies (46%–92%) [5, 36, 38, 43–46], likely reflecting differences in study populations, particularly the inclusion of dogs presenting with ruptured tumors or hemoabdomen.

In the present study, 59.3% (32/54) of dogs with hemoabdomen were diagnosed with HSA, and a strong association between these variables was identified, supporting previous observations that this neoplasm is particularly prone to rupture and bleed prior to diagnosis [9, 42, 47]. However, a substantial proportion of dogs with HSA in the present cohort did not present with hemoabdomen at diagnosis, and several cases were identified incidentally during ultrasonographic evaluation or presented only with abdominal signs. These findings highlight that, although abdominal bleeding should raise strong clinical suspicion for splenic HSA, its absence does not exclude this diagnosis.

In addition to the association between HSA and hemoabdomen, several statistically significant relationships identified in this study further support the complex clinicopathological presentation of splenic disease in dogs, particularly the association between incidental detection and non‐neoplastic diagnoses, as well as the frequent coexistence of multiple lesions within the same spleen.

Hemorrhagic splenic lesions represent an important diagnostic challenge in veterinary pathology. Previous studies have emphasized the frequent coexistence of splenic hematoma and HSA, noting that hemorrhagic lesions may partially or completely obscure underlying malignant vascular tumors [2, 18, 31, 39]. In such cases, incomplete sampling or limited histological evaluation may lead to underdiagnosis of HSA. These findings support current recommendations advocating extensive sampling of hemorrhagic splenic masses and, when indicated, the use of immunohistochemistry to improve diagnostic accuracy.

Interestingly, no significant association between splenic hematoma and HSA was identified in the present study. This finding likely reflects the high proportion of primary hematomas within the cohort that were not associated with underlying neoplasia, which may dilute the apparent relationship between hematoma formation and malignancy. Therefore, although hematomas may coexist with splenic neoplasia, their presence alone should not be considered a reliable predictor of HSA.

One of the most notable findings of this study was the high prevalence of splenitis, which represented 54.6% (154/282) of non‐neoplastic cases. This proportion greatly exceeds those reported in previous studies, where inflammatory splenic lesions typically represent less than 8% of diagnoses [1, 7, 8, 37, 48]. Several factors may explain this discrepancy.

First, regional epidemiological factors may contribute to increased stimulation of the spleen. Dogs in Colombia are frequently exposed to a variety of vector‐borne and infectious agents, including *Ehrlichia*, *Anaplasma*, and *Leishmania*, with reported seroprevalence rates exceeding 30%–70% in some regions [49]. Although special stains or ancillary tests were not performed to identify infectious agents in the present study, chronic antigenic stimulation associated with these pathogens could contribute to reactive splenitis.

Second, methodological and diagnostic differences may have influenced the recorded prevalence. In the present cohort, splenitis was documented whenever histological evidence of inflammation was identified, even when other lesions were present concurrently. In addition, hemorrhagic splenitis represented the most frequent inflammatory pattern (46.8%, 72/154). In some diagnostic settings, similar lesions might instead be interpreted as secondary hemorrhage rather than primary inflammatory disease, reflecting the absence of universally accepted histopathological criteria for distinguishing these processes. Consequently, interobserver variability among pathologists may partially explain differences between studies. These findings highlight the importance of interpreting splenic lesions within their broader clinical and epidemiological context, as multiple pathological processes may coexist and influence the final histopathological diagnosis.

Another important observation was the frequent occurrence of concurrent histopathological diagnoses within the same spleen. Unlike many neoplastic diseases that tend to present as single dominant lesions, non‐neoplastic splenic cases often represent overlapping or sequential pathological processes. This complexity highlights an important diagnostic consideration: Evaluation of limited tissue samples may fail to detect clinically relevant lesions present elsewhere in the organ. For this reason, systematic histological examination of multiple sections from different regions of the organ is recommended to improve diagnostic accuracy.

The relatively high proportion of incidentally detected splenic lesions in this cohort also raises important clinical considerations. Approximately one‐third of the cases were discovered incidentally (143/424), and the majority of these lesions were non‐neoplastic (74.8%, 107/143). Similar findings have been reported in studies evaluating incidentally detected splenic lesions, where benign diagnoses represented most cases [9, 14]. These results suggest that not all splenic lesions detected during imaging necessarily require immediate splenectomy, and careful clinical evaluation is warranted to balance the potential benefits and risks of surgical intervention. Future studies comparing clinical outcomes between surgically and conservatively managed dogs with incidental splenic findings would be valuable.

Finally, several limitations should be acknowledged. The retrospective design and reliance on submission form data may have introduced selection and information biases. In addition, cases were obtained from three referral histopathology laboratories rather than from a population‐based cancer registry; therefore, the findings should not be interpreted as incidence estimates for the general canine population in Colombia. Differences in referral patterns, case submission, and completeness of clinical records among centers may also have influenced the study population. Histopathological evaluation was based on submitted tissue samples rather than standardized whole‐organ protocols in all cases, potentially affecting lesion detection. Furthermore, immunohistochemistry was not routinely available, which may have limited the precise classification of some mesenchymal tumors, particularly when differentiating solid HSA from undifferentiated or stromal sarcomas.

## 5. Conclusions

This study provides epidemiological insights into splenic lesions in dogs undergoing splenectomy in a regional clinical population from Colombia. Non‐neoplastic lesions predominated, and splenitis represented an unexpectedly high proportion of diagnoses, suggesting that regional epidemiological factors may influence the spectrum of splenic disease. The frequent occurrence of concurrent histopathological lesions further highlights the diagnostic complexity of splenic pathology and the importance of thorough histological evaluation. These findings also underscore the need for greater consensus between clinicians and pathologists regarding diagnostic criteria for splenic lesions and standardized sampling approaches to optimize histopathological assessment. Furthermore, incomplete clinical information provided at the time of submission may limit diagnostic interpretation, highlighting the importance of comprehensive clinical–pathological communication.

## Author Contributions

All authors included herein made substantial contributions, as follows: Hathali de las Mercedes Sanchez Mata: conception and design of the study, acquisition and interpretation of data, graphing, and drafting the manuscript for important intellectual content. Janeth Agudelo Quintero: conception and design of the study, interpretation of data, and revising the manuscript for important intellectual content. Diego Alfonso Aranzazu Taborda: conception and design of the study, laboratory analysis, pathological interpretation, and revising the manuscript for important intellectual content. David Alzate Velásquez: acquisition and interpretation of data and revising of the manuscript for important intellectual content. Julián David Muñoz Duque: conception and design of the study, acquisition and interpretation of data, laboratory analysis, pathological interpretation, drafting, and revising the manuscript for important intellectual content.

## Funding

The authors received no financial support for the research, authorship, and/or publication of this article.

## Disclosure

All authors gave the final approval of the version to be submitted.

## Conflicts of Interest

The authors declare no conflicts of interest.

## Supporting Information

Additional supporting information can be found online in the Supporting Information section.

## Supporting information


**Supporting Information** Supporting Table S1. Detailed non‐neoplastic splenic histopathological diagnoses in dogs. Supporting Table S2. Secondary histopathological findings associated with non‐neoplastic splenic diagnoses in dogs. Supporting Table S3. Detailed neoplastic splenic histopathological diagnoses in dogs. Supporting Table S4. Secondary histopathological findings associated with splenic neoplasms in dogs.

## Data Availability

The data that support the findings of this study are available on request from the corresponding author. The data are not publicly available due to privacy or ethical restrictions.
