# Supplementary material for: Splenic Lesions in Dogs: A Retrospective Epidemiological and Pathological Study
Source: Vet Med Int. 2026 Aug 21;2026:9086933. doi: 10.1155/vmi/9086933 (PMC13498858; doi:10.1155/vmi/9086933)
Supplement: Supplementary file 1 [file VMI-2026-9086933-s001.docx]

**Supplementary Tables**

**Supplementary Table S1.** Detailed non-neoplastic splenic histopathological diagnoses in dogs.

| **Non-neoplastic diagnosis &** | **Cases n (% of 282 dogs)+** | **Median age, years (range)** | **Sex (F/M)** | **Most affected breeds** | **Common macroscopic pattern** | **Common concurrent morphological diagnosis** | **Common associated histopathological findings** |
| --- | --- | --- | --- | --- | --- | --- | --- |
| **Splenitis** | 154 (54.61%) | 9.5ᵃ (<1-16) | 81/73 | Crossbreed 27.9% (43/154); Beagle 7.8% (12/154); Pit Bull Terrier 7.8% (12/154); Bulldog 6.5% (10/154); Labrador Retriever 5.8% (9/154) | Single 87/154; Multiple 25/154; Splenomegaly 22/154 | SH 28.6% (44/154); NLH 10.4% (16/154); EMH 10.4% (16/154); SN 10.4% (16/154); Telangiectasia 5.8% (9/154); Hemosiderosis 5.2% (8/154) | EMH 83.1% (128/154); Hemorrhage 79.9% (123/154); LTC* 76% (117/154); SH 29.9% (46/154) |
| **SH** | 95 (33.7%) | 10.1ᵇ (4-16) | 37/41 | Crossbreed 20% (19/95); Pit Bull Terrier 8.4% (8/95); Golden Retriever 7.4% (7/95); Beagle 7.4% (7/95); Bulldog (6.3%, 6/95) | Single 73/95; Multiple 9/95 | Splenitis 46.3% (44/95); NLH 7.4% (7/95); EMH 7.4% (7/95); Telangiectasia 5.3% (5/95); SN 3.2% (3/95) | Hemorrhage 84.2% (80/95); EMH 68.4% (65/95); Inflammation 63.2% (60/95); LTC 56.8% (54/95) |
| **NLH** | 63 (22.3%) | 8.9ᶜ (<1-15) | 28/35 | Crossbreed 33.3% (21/63); Poodle 11.1% (7/63); Shih Tzu 7.9% (5/63); Beagle 6.3% (4/63) | Single 54/63; Multiple 7/63 | Splenitis 25.4% (16/63); SH 11.1% (7/63); EMH 6.3% (4/63); SN 4.8% (3/63) | Hemorrhage 65% (41/63); EMH 54% (34/63); Inflammation 52.4% (33/63) |
| **EMH** | 30 (10.6%) | 9.6ᵈ (<1-15) | 17/13 | Crossbreed 23.3% (7/30); Poodle 13.3% (4/30); Pit Bull Terrier 10% (3/30); Bulldog 10% (3/30); Schnauzer (6.7%; 2/30) | Single 14/30; Multiple 9/30 | Splenitis 53.3% (16/30); SH 23.3% (7/30); NLH 13.3% (4/30); OTH 10% (3/30); SN 10% (3/30) | LTC 73.3% (22/30); Hemorrhage 66.7% (20/30); Inflammation 56.7% (17/30) |
| **SN** | 27 (9.6%) | 8.4 (1-14) | 12/15 | Crossbreed 33.3% (9/27); Bulldog 22.2% (6/27); Beagle 7.4% (2/27); Pit Bull Terrier 7.4% (2/27) | Single 12/27; Splenomegaly 7/27; Multiple 4/27 | Splenitis 59.3% (16/27); SH 11.1% (3/27); EMH 11.1% (3/27); NLH 11.1% (3/27); SH 11.1% (3/27) | EMH 70.4% (19/27); Hemorrhage 63% (17/27); Inflammation 63% (17/27); LTC 63% (17/27) |
| **Hemosiderosis** | 16 (5.7%) | 9.5ᵉ (1-16) | 10/6 | Crossbreed 18.8% (3/16); Beagle 12.5% (2/16); Pug 12.5% (2/16) | Single 9/16; Splenomegaly 3/16; Multiple 3/16 | Splenitis 50% (8/16); SC 18.8% (3/16); SN 12.5% (2/16); OTH 12.5% (2/16); LA 12.5% (2/16) | LTC 93.8% (15/16); Inflammation 56.3% (9/16); Hemorrhage 43.8% (7/16) |
| **OTHᶠ** | 14 (5%) | 9.1ᵍ (3-13) | 10/4 | Crossbreed 14.3% (2/14); Pug 14.3% (2/14); Schnauzer 14.3% (2/14) | Single 9/14; Multiple 2/14 | Splenitis 35.7% (5/14); SH 21.4% (3/14); EMH 21.4% (3/14); Hemosiderosis 14.3% (2/14) | LTC 100% (14/14); EMH 78.6% (11/14); Inflammation 64.3% (9/14); Hemorrhage 57.1% (8/14) |
| **Telangiectasia** | 13 (4.6%) | 10.5 (5-15) | 7/6 | Poodle 15.4% (2/13); Pit Bull Terrier 15.4% (2/13) | Single 10/13 | Splenitis 69.2% (9/13); SH 38.5% (5/13) | Inflammation 92.3% (12/13); Hemorrhage 84.6% (11/13); EMH 76.9% (10/13) |
| **HN** | 8 (2.8%) | 10.1 (6-13) | 1/7 | Labrador Retriever 37.5% (3/8); Beagle 25% (2/8) | Single 4/8; Splenomegaly 2/8; Multiple 2/8 | Splenitis 25% (2/8); SH 12.5% (1/8); NLH 12.5% (1/8); SN 12.5% (1/8) | Necrosis 100% (8/8); Hemorrhage 100% (8/8); EMH 62.5% (5/8) |
| **SH** | 7 (2.48%) | 9.7 (7-14) | 3/4 | Crossbreed 28.6% (2/7) | Multiple 3/7; Single 2/7 | SN 42.9% (3/7); Splenitis 28.6% (2/7) | Hemorrhage 100% (7/7); EMH 85.7% (6/7); LTC 57.1% (4/7) |
| **LA** | 5 (1.8%) | 9.2 (5-12) | 4/1 | Crossbreed 20% (1/5); Poodle 20% (1/5); Golden Retriever 20% (1/5); Boston Terrier 20% (1/5); Husky 20% (1/5) | Single 4/5 | Splenitis 40% (2/5); SH 40% (2/5); Hemosiderosis 40% (2/5) | LTC 100% (5/5); EMH 100% (5/5); Hemorrhage 60% (3/5) |
| **SC** | 5 (1.8%) | 9.5^h^ (5-14) | 1/4 | Crossbreed 20% (1/5); Belgian Shepherd 20% (1/5); Beagle 20% (1/5); Golden Retriever 20% (1/5); Pit Bull Terrier 20% (1/5) | Multiple 3/5; Single 2/5 | Hemosiderosis 60% (3/5); LD 20% (1/5); EMH 20% (1/5) | LTC 100% (5/5); EMH 60% (3/5) |
| **LD** | 5 (1.8%) | 8.5^i^ (<1-14) | 1/4 | Crossbreed dogs 20% (1/5); Pit Bull Terrier 20% (1/5); Shar-Pei 20% (1/5); Labrador Retriever 20% (1/5); German shepherd 20% (1/5) | Single (3/5); Splenomegaly (1/5) | Splenitis 40% (2/5); SC 20% (1/5); Hemosiderosis 20% (1/5); SH 20% (1/5) | LTC 100% (5/5); Inflammation 80% (4/5); EMH 60% (3/5) |
| **Fibrosis** | 5 (1.8%) | 10.6 (8-13) | 4/1 | Schnauzer 60% (3/5); Labrador Retriever 20% (1/5); Crossbreed 20% (1/5) | Single (2/5); Splenomegaly (2/5) | NLH 40% (2/5); Splenitis 20% (1/5); SH 20% (1/5) | EMH 100% (5/5); Hemorrhage 80% (4/5); LTC 80% (4/5) |
| **HI** | 1 (0.4%) | 11.0 | M | German Shepherd | Multiple (1/1) | N/A | Necrosis; Hemorrhage; Inflammation |
| **Perisplenitis** | 1 (0.4%) | 12.0 | F | Beagle | Single (1/1) | Splenitis 100% (1/1) | Necrosis; Hemorrhage; EMH; Inflammation |

**Abbreviations:** Single = single mass; Multiple = multiple masses; NLH = nodular lymphoid hyperplasia; EMH = extramedullary hematopoiesis; SH = splenic hematoma; SN = siderotic nodules; OTH = other types of hyperplasia; SC = splenic congestion; LA = lymphoid atrophy; LD = lymphoid depletion; LTC = lymphoid tissue changes; HN = hemorrhagic necrosis; HI = hemorrhagic infarction; N/A = not applicable.
 **Notes**

Supplementary Table S1 provides the complete dataset of non-neoplastic splenic diagnoses included in the study.

& Because multiple morphological processes could occur within the same spleen, diagnoses were not mutually exclusive and more than one diagnosis could be recorded per case.

+ Percentages were calculated relative to the total number of dogs with non-neoplastic splenic lesions (n = 282).

*****Lymphoid tissue changes include white pulp alterations such as hyperplasia, atrophy, depletion, or neoplasia when reported in the original histopathology reports.

Splenic hematoma listed in this table corresponds only to cases diagnosed as primary non-neoplastic lesions

ᵃ In three cases the age was not reported and one dog was <1 year old.

ᵇ The age was not reported in one case.

ᶜ In two cases the age was not reported and one dog was <1 year old.

ᵈ The age was not reported in one case and one dog was <1 year old.

ᵉ The age was not reported in a case

ᶠ In some cases megakaryocytic, reticulo-histiocytic, lymphoid, or reactive hyperplasia were observed

ᵍ The age was not reported in a case

^h^ The age was not reported in a case

^i^ The age was not reported in a case and a report was < 1-year-old

**Table S2.** Secondary histopathological findings associated with non-neoplastic splenic diagnoses in dogs

| **Histopathological Diagnosis** | **Other histopathological findings** | | | | | | | | |
| --- | --- | --- | --- | --- | --- | --- | --- | --- | --- |
|  | **Necrosis** | **Hemorrhage** | **Extramedullary hematopoiesis** | **Inflammation** | **Hematoma** | **Siderotic nodules** | **Lymphoid tissue changesᵃ** | **Central arteriolar thickening** | **Total of findings** |
| **Splenitis** | 26 | 123 | 128 | 154 | 46 | 25 | 117 | 17 | 636 |
| **Splenic hematoma** | 17 | 80 | 65 | 60 | 95 | 11 | 54 | 6 | 388 |
| **Lymphoid nodular hyperplasia** | 7 | 41 | 34 | 33 | 8 | 8 | 63 | 6 | 200 |
| **Extramedullary hematopoiesis** | 2 | 20 | 30 | 17 | 9 | 8 | 22 | 4 | 112 |
| **Siderotic nodules** | 8 | 17 | 19 | 17 | 3 | 27 | 17 | 2 | 110 |
| **Hemosiderosis** | 0 | 7 | 12 | 9 | 1 | 2 | 15 | 0 | 46 |
| **Other types of hyperplasia** ^g^ | 1 | 8 | 11 | 9 | 3 | 1 | 14 | 0 | 47 |
| **Telangiectasia** | 1 | 11 | 10 | 12 | 6 | 3 | 7 | 4 | 54 |
| **Hemorrhagic necrosis** | 8 | 8 | 5 | 3 | 1 | 2 | 3 | 0 | 30 |
| **Hemorrhage** | 1 | 7 | 6 | 2 | 0 | 3 | 4 | 0 | 19 |
| **Lymphoid atrophy** | 0 | 3 | 5 | 2 | 2 | 0 | 5 | 1 | 18 |
| **Splenic congestion** | 0 | 1 | 3 | 0 | 0 | 0 | 5 | 0 | 9 |
| **Lymphoid depletion** | 2 | 2 | 3 | 4 | 1 | 0 | 5 | 0 | 17 |
| **Fibrosis** | 0 | 4 | 5 | 3 | 1 | 0 | 4 | 0 | 17 |
| **Splenosis** | 0 | 2 | 2 | 1 | 0 | 0 | 2 | 0 | 7 |
| **Hemorrhagic infarction** | 1 | 1 | 0 | 1 | 0 | 0 | 0 | 0 | 3 |
| **Perisplenitis** | 1 | 1 | 1 | 1 | 0 | 0 | 0 | 0 | 4 |

ᵃ Lymphoid tissue changes include hyperplasia, depletion, or atrophy of the splenic white pulp.

ᵇIn some cases megakaryocytic, reticulo-histiocytic, lymphoid, or reactive hyperplasia were observed

**Table S3.** Detailed neoplastic splenic histopathological diagnoses in dogs.

| **Neoplastic diagnosis** | **Cases n (% of 142 dogs)** | **Median age, years (range)** | **Sex (F/M)** | **Most affected breeds** | **Common macroscopic pattern** | **Common concurrent non-neoplastic diagnosis** | **Common associated histopathological findings** |
| --- | --- | --- | --- | --- | --- | --- | --- |
| **Malignant tumors (n = 130)** | | | | | | | |
| **Hemangiosarcoma (HSA)**ᵃ | 90 (63.4%)ᵇ | 10.6 (5-17) | 42/48 | Crossbreed 34.4% (31/90); Schnauzer 15.6% (14/90); Golden Retriever 6.7% (6/90); Beagle 5.6% (5/90); Yorkshire Terrier 4.4% (4/90); German Shepherd (4.4%, 4/90) | Single (52/90); multiple (24/90) | SH 3.3% (3/90); EMH 1.1% (1/90); HN 1.1% (1/90) | Hemorrhage 78.9% (71/90); Necrosis 56.7% (51/90); Hematoma 40% (36/90); Inflammation 33.3% (30/90); EMH 28.9% (26/90) |
| **Lymphoma**ᶜ | 20 (14.1%) | 9.3 (4-14) | 11/9 | Crossbreed 45% (9/20); Bulldog 15% (3/20); Rottweiler 10% (2/20) | Splenomegaly (6/20); single (5/20); multiple (4/20); nodular (2/20) | Splenitis 5% (1/20); EMH 5% (1/20) | LTC* 100% (20/20); Hemorrhage 65% (13/20); Necrosis 40% (8/20); EMH 30% (6/20); Inflammation 25% (5/20) |
| **Undifferentiated sarcoma**ᵈ | 8 (5.6%) | 10.0 (7-12) | 3/5 | Pit Bull Terrier 25% (2/8); Golden Retriever 12.5% (1/8); Labrador Retriever 12.5% (1/8); Schnauzer 12.5% (1/8); Shih Tzu 12.5% (1/8); Pomeranian 12.5% (1/8); Yorkshire Terrier 12.5% (1/8) | Single (8/8) | Splenitis 12.5% (1/8); EMH 12.5% (1/8) | Hemorrhage 75% (6/8); Inflammation 75% (6/8); EMH 37.5% (3/8); Necrosis 37.5% (3/8); LTC 12.5% (1/8) |
| **Fibrosarcoma** | 4 (2.8%) | 9.0 (7-10) | 4/0 | Crossbreed 50% (2/4); Pit Bull Terrier 25% (1/4); Basset Hound 25% (1/4) | Single (4/4) | Splenitis 25% (1/4) | LTC 50% (2/4); Necrosis 50% (2/4) |
| **Histiocytic sarcomaᵉ** | 4 (2.8%) | 10.5 (6-13) | 1/3 | Crossbreed 50% (2/4); Bernese Mountain Dog 25% (1/4); Boston Terrier 25% (1/4) | Single (2/4); multiple (1/4) | N/A | Hemorrhage 75% (3/4); necrosis 75% (3/4); Inflammation 50% (2/4); EMH 50% (2/4) |
| **Liposarcoma**ᶠ | 2 (1.4%) | 5.0 (4-6) | 1/1 | Doberman 50% (1/2); Weimaraner 50% (1/2) | Single (1/2) | N/A | Hemorrhage 100% (2/2); Necrosis 50% (1/2); EMH 50% (1/2); LTC 50% (1/2) |
| **Myxosarcoma** | 1 (0.7%) | 12.0 | 1/0 | Pit Bull Terrier | Single | N/A | N/A |
| **Mast cell tumor** | 1 (0.7%) | Not reported | 0/1 | Crossbreed | Single | N/A | Necrosis (100%, 1/1); Hemorrhage 100% (1/1); EMH 100% (1/1) |
| **Benign tumors (n = 12)** | | | | | | | |
| **Myelolipoma** | 8 (5.6%) | 10.0 (8-13) | 2/6 | Labrador Retriever 25% (2/8); Crossbreed 25% (2/8) | Single (4/8); multiple (2/8) | SH 25% (2/8); Splenitis 25% (2/8); NLH 12.5% (1/8); SN 12.5% (1/8) | Inflammation 75% (6/8), EMH 75% (6/8); Hemorrhage 50% (4/8); LTC 50% (4/8) |
| **Plasmacytoma**ᵍ | 3 (2.1%) | 11.0 (9-14) | 0/3 | Pit Bull Terrier 66.6% (2/3); Golden Retriever 33.3% (1/3) | Multiple (2/3); Splenomegaly (1/3) | Splenitis 33.3% (1/3); SN 33.3% (1/3); EMH 33.3% (1/3) | Hemorrhage 100% (3/3); Inflammation 66.6% (2/3); EMH 66.6% (2/3) |
| **Cavernous hemangioma** | 1 (0.7%) | 11.0 | 1/0 | Chihuahua | Single (1/1) | EMH 100% (1/1) | Hemorrhage 100% (1/1); EMH 100% (1/1) |

**Abbreviations:** Single = single mass; Multiple = multiple masses; Nodular = nodular pattern; SH = splenic hematoma; EMH = extramedullary hematopoiesis; HN = hemorrhagic necrosis; N/A = not applicable; NLH = nodular lymphoid hyperplasia; SN = siderotic nodules; LTC = lymphoid tissue changes.

**Notes**

*Lymphoid tissue changes include white pulp alterations such as hyperplasia, atrophy, depletion, or neoplasia when reported.

ᵃ Three cases were consistent with hemangiosarcoma and require more specific tests to confirm the diagnosis

ᵇ In two cases, the age was not reported.

ᶜ One case were consistent with lymphoma and require more specific tests to confirm the diagnosis

ᵈ Cases previously diagnosed as fibrohistiocytic nodules (n = 3) and malignant fibrous histiocytoma (n = 1) were reclassified as undifferentiated sarcoma according to current classification criteria. Immunohistochemistry was not available, which may represent a potential source of misclassification.

ᵉ Three cases were consistent with histiocytic sarcoma and require more specific tests to confirm the diagnosis

ᶠ One case were consistent with lymphosarcoma and require more specific tests to confirm the diagnosis

ᵍ They were categorized as indeterminate.

**Table S4.** Secondary histopathological findings associated with splenic neoplasms in dogs

| **Histopathological Diagnosis** | **Other histopathological findings** | | | | | | | | |
| --- | --- | --- | --- | --- | --- | --- | --- | --- | --- |
|  | **Necrosis** | **Hemorrhage** | **Extramedullary hematopoiesis** | **Inflammation** | **Hematoma** | **Siderotic nodules** | **Lymphoid tissue changes**ᵇ | **Central arteriolar thickening** | **Total of findings** |
| **Hemangiosarcomaᵃ** | 51 | 71 | 26 | 30 | 36 | 4 | 5 | 0 | 223 |
| **Lymphomaᶜ** | 8 | 13 | 6 | 5 | 3 | 1 | 20 | 0 | 37 |
| **Undifferentiated sarcomaᵈ** | 5 | 6 | 3 | 6 | 2 | 0 | 2 | 0 | 24 |
| **Fibrosarcoma** | 2 | 1 | 0 | 1 | 0 | 0 | 2 | 1 | 7 |
| **Histiocytic sarcomaᵉ** | 3 | 3 | 2 | 2 | 0 | 0 | 1 | 1 | 12 |
| **Liposarcoma**ᶠ | 1 | 2 | 1 | 0 | 0 | 0 | 1 | 0 | 5 |
| **Myxosarcoma** | 0 | 0 | 0 | 0 | 0 | 0 | 0 | 0 | 0 |
| **Mast cell tumor** | 1 | 1 | 1 | 0 | 0 | 0 | 0 | 0 | 3 |
| **Myelolipoma** | 3 | 4 | 6 | 6 | 2 | 2 | 4 | 0 | 27 |
| **Plasmacytoma**ᵍ | 1 | 3 | 2 | 2 | 1 | 1 | 1 | 0 | 11 |
| **Cavernous hemangioma** | 0 | 1 | 1 | 0 | 0 | 0 | 0 | 0 | 2 |

ᵃ Three cases were consistent with hemangiosarcoma and require more specific tests to confirm the diagnosis

ᵇ Lymphoid tissue changes include alterations of the splenic white pulp such as hyperplasia, depletion, atrophy, or neoplastic infiltration.

ᶜ One case was consistent with lymphoma and require more specific tests to confirm the diagnosis

ᵈ Cases previously diagnosed as fibrohistiocytic nodules (n = 3) and malignant fibrous histiocytoma (n = 1) were reclassified as undifferentiated sarcoma according to current classification criteria. Immunohistochemistry was not available, which may represent a potential source of misclassification.

ᵉThree cases were consistent with histiocytic sarcoma and require more specific tests to confirm the diagnosis.

ᶠ One case was consistent with lymphosarcoma and require more specific tests to confirm the diagnosis.

ᵍ They were categorized as indeterminate
